# Out-of-Home-Care Rates among Indigenous and Non-Indigenous Children in Countries With Histories of Settler Colonialism

**DOI:** 10.1177/10775595251411524

**Published:** 2025-12-23

**Authors:** Martin Eiermann, Mikkeline Munk Nielsen, Christopher Wildeman, Peter Fallesen

**Affiliations:** 1 5228 University of Wisconsin - Madison, Madison, WI, USA; 2 519195 ROCKWOOL Foundation, Copenhagen, Denmark; 3 4321 University of Copenhagen, Copenhagen, Denmark; 4 3065 Duke University, Durham, NC, USA; 5 7675 Stockholm University, Stockholm, Sweden

**Keywords:** child protective services, ethnic minority populations, foster children, population

## Abstract

Indigenous children in settler-colonial societies have historically been exposed to frequent family separation; yet contemporary family separation through out-of-home-care (OOHC) remains understudied. We analyzed annual OOHC rates among indigenous and non-indigenous children (2010–2023) in four countries: Australia, United States, Denmark, and Kalaallit Nunaat (Greenland). Data sources included national child welfare databases and population registers. We computed observed annual rates and generated age-standardized rates using parametric bootstrap approaches with Generalized Additive Models. We found that indigenous children experienced substantially higher OOHC rates across all countries. Annual rates ranged from around 1.5% (US) to around 6% (Australia, Greenland) among indigenous children, versus 0.6–0.9% among non-indigenous children. Risk ratios were highest in Australia (10.1–11.4) and lowest in the US (1.5–1.9). Our findings demonstrate that indigenous children remain disproportionately exposed to OOHC, with substantial cross-national variation in magnitude and age patterns that likely reflects different policy environments and child welfare practices.

## Introduction

The removal of children from their families is one of the most high-stakes interventions that Child Protective Services (CPS) have at their disposal to mitigate the exposure of vulnerable children to environments deemed harmful. Yet it is also controversial, especially when out-of-home-care (OOHC) placements involve indigenous children.

Historically, the forcible separation of indigenous children from their families has been a central element of government policy in settler colonialist societies. In the United States, the Indian Schools system of the late 19th century and the subsequent Indian Adoption Project (1958–1967) facilitated the removal of thousands of Native American children from their families and communities (Jacobs, 2014). In Australia, government policy between 1910 and 1970 likewise authorized the forcible removal of Aboriginal and Torres Strait Islander children from their families, creating what are now referred to as the “stolen generations” of indigenous children (Krakouer et al., 2018). In Canada, the forcible removal of indigenous children by child welfare agencies culminated in the so-called “Sixties Scoop”, when an estimated 20,000 native children were placed in predominantly White foster families or adoption homes between the 1950s and 1980s (Sinclair, 2007). In Kalaallit Nunaat (Greenland), 22 “experiment children” were removed from their families and educated in Denmark as “cultural ambassadors” in the 1950s, and several hundred Greenlandic (Kalaallit Inuit) children were adopted by Danish parents in the 1950s–70s on a questionable legal basis and at great personal and cultural expense (Thorleifsen et al., 2020; Tróndheim, 2010).

These historical legacies cast shadows over discussions of OOHC placements in the present, which are alternatively seen as welfarist interventions that can remove children from environments that do not allow them to thrive, as sources of new vulnerabilities in the lives of already-disadvantaged children, or as the latest chapters in a long history of state policies that separate indigenous children from their ancestral lands and communities (Courtney & Hook, 2017; Edwards et al., 2023; Thorleifsen et al., 2020; Valentine & Gray, 2006).

However, empirical knowledge of contemporary OOHC among indigenous children remains limited and varies considerably across countries. In the US, Canada, Australia, New Zealand, and Greenland, prior work has shown that indigenous children experience particularly high annual and cumulative rates of OOHC (Edwards et al., 2023; Stanley & de Froideville, 2020; Tilbury, 2009; Trocmé et al., 2004; Wildeman et al., 2025; Yi et al., 2020). But inconsistent definitions of indigeneity and the lack of age-standardized estimates impede precise estimates of annual rates and comparative analyses.

We update and extend existing studies by estimating annual rates of OOHC, as well as disparities in OOHC between indigenous and non-indigenous children, in four countries with settler-colonial histories and/or large indigenous populations: the US, Australia, Denmark, and Greenland. Specifically, these analyses (1) facilitate cross-country comparisons by estimating age-standardized rates of OOHC; (2) update prior estimates of indigenous OOHC rates in the US and Australia, and (3) provide the first-ever estimates of such rates in Denmark and Greenland.

## Contemporary Patterns of OOHC among Indigenous Children

Prior research on indigenous OOHC is limited. Comparatively good estimates exist in Australia, where official government reports show that around 5.7% of indigenous children experienced OOHC during the 2022-23 year, compared to 0.5% of non-indigenous children; and OOHC admission rates were highest for very young children under age 1 (Australian Institute of Health and Welfare, 2025a). More than half of indigenous children in OOHC (63%) were living with relatives or kin, and around three quarters (72%) had been in continuous OOHC for more than two years.

In the US, research has shown that American Indian/Alaska Native (AIAN) children have the highest cumulative risk of foster care placement by age 18 of any racial group (11.4% in 2016, compared to 5.0% among White children) and have also experienced the greatest percentage point increase in cumulative risk since 2004 (Yi et al., 2020). Recent studies show that around 33,000 indigenous children experience OOHC in any given year (Edwards et al., 2023). However, precise estimates are complicated by definitional ambiguities. The count of 33,000 annual cases includes multiracial children (“AIAN alone or in combination with any other racial or ethnic category”), but other recent studies only count single-race and non-Hispanic AIAN children (Edwards et al., 2021; Yi et al., 2020). Matching those alternative definitions to population denominators—which is required to obtain annual OOHC rates—is also not straightforward, since commonly used year-, age-, and race-specific population estimates from the National Institutes of Health’s Surveillance, Epidemiology and End Results (SEER) program use bridged racial categories that assign a single race to multiracial children.

In Greenland, very few systematic analyses of OOHC among Greenlandic Inuit children exist, and the requisite data are not collected as part of the official statistics. A review by Jensen (2021) found that 4.2% to 7.0% of Greenlander children experienced OOHC in a given year, with differences in the estimates due to differences in measurement strategies. To our knowledge, knowledge on indigenous OOHC in Denmark—that is, OOHC among children of Greenlander ancestry who were born or now reside in Denmark—is limited to a single report, which found that those children were 5–7 times more likely to experience OOHC than children with Danish parents (VIVE, 2022). As a result, while the *historical* fact of family separation is well-established, comparatively little is known about *contemporary* standardized rates of OOHC among indigenous children.

## Defining Indigeneity

One central challenge for researchers is definitional. Establishing who counts as “indigenous” is fraught with difficulty (Ring, 2003); and the socio-historical contexts that influence debates about identity and belonging differ across societies. Such debates are in part about qualifying characteristics (such as ancestry or cultural ties to First Nations) and in part about the recognized arbiters of indigenous status. Individuals may self-identify as indigenous or be recognized (or not recognized) as indigenous by tribal or First Nations authorities, by non-tribal administrative agencies, or under state and federal law regardless of their self-identification. Additionally, in the United States, Pacific Islanders, Hispanic children, and multiracial children are sometimes but not always included in administrative tallies of indigenous populations.

We pragmatically adopted definitions of indigeneity that were informed by national debates and child welfare practice. In Australia, indigeneity is self-reported by children or their primary caregivers at the local level and is understood to include children with Aboriginal or Torres Strait Islander ancestry. Territorial and national welfare agencies defer to those local self-reports when classifying children. We adopted the same position.

In the US, race and ethnicity are generally self-reported by caregivers (at younger ages) or the child (at older ages), but there is no agreed-upon definition of indigeneity. Historically, administrative agencies used tribal enrollment as a measure of indigeneity or, more recently, relied on mutually exclusive ethnoracial categories and counted only non-Hispanic single-race AIAN children as indigenous. Increasingly, however, indigeneity has been defined to include multi-racial and Hispanic persons who also identify as AIAN (Brown, 2020; Snipp, 2003). Government agencies and researchers now make different decisions regarding (a) the inclusion of Pacific Islander/Hawaiian Native children in the indigenous category, (b) the inclusion of multiracial children, and (c) the inclusion of Hispanic children. Both definitions have been used in prior research, although recent research practice has tended to operationalize the newer and more expansive definition of indigeneity (Edwards et al., 2023). We followed this trend (which also aligns with evolving cultural conceptions of ethno-racial identity that emphasize the concurrent membership of mixed-populations in multiple ethnic and racial groups, see Nobles, 2000) by counting all children as indigenous who self-identified as AIAN. This included multiracial children who identified as part-indigenous and children who either did, or did not, identify as Hispanic. This is a consequential choice, which we discuss in the Limitations section. We additionally present core findings from a different specification (counting only single-race, non-Hispanic AIAN children as indigenous) in a footnote in the Results section.

In Greenland, the majority of the population is native born and indigenous and part of the Inuit ethnic group, also known as Greenlandic/Kalaallit Inuit or Greenlanders. According to data from Statistics Greenland’s Data Bank, only 5% of under-18 residents are not native-born, and the likelihood of non-native-born children being placed in OOHC in Greenland is exceedingly low. (Greenlander children may live in Denmark and experience OOHC there; however, Greenland-based experts confirmed that non-indigenous children from Denmark or other Nordic countries, which makes up the vast majority of non-indigenous child population, are not placed in OOHC with Greenlander families in Greenland.) Accordingly, we counted all children in Greenland as indigenous.

In Denmark, we used administrative data on parental birthplace from the Danish Population Register (*Befolkningen,* BEF)—using administrative birthplace codes—to identify children of Greenlander ancestry, which we defined as having at least one parent born in Greenland and then counted as indigenous. We adopted this approach because Danish authorities do not collect self-reported data on indigeneity and because Greenlander identity is primarily understood in Denmark through the lens of ancestral birthplace. We counted children with at least one Greenland-born parent as indigenous to recognize the possibility of mixed-race identities in Denmark, which aligns our Danish measure of indigeneity with our US-based approach and also reflects contemporary approaches to the study of Greenlander children in Denmark in a child welfare context (VIVE, 2022).

We decided to compare indigenous to non-indigenous children, setting aside other potential reference populations (e.g., White children in the US). We made this decision because the US is the only country in our sample that systematically collects child welfare data by race. In Australia, for example, authorities only record a child’s indigeneity but do not record the race (e.g. White or Asian) of non-indigenous children. Comparing indigenous children to all non-indigenous children makes cross-national comparisons possible and allows us to focus on indigeneity as a core dimension of OOHC, without wading into nationally-specific debates about more complex racial hierarchies.

## Defining Out-of-Home-Care

Definitions of OOHC can vary across countries and are sometimes operationalized inconsistently even within countries. The countries included in this study have adopted definitions of OOHC that are largely, but not wholly, aligned. Each country considers OOHC to include (a) placements in the homes of non-kinship caregivers (“foster parents”), who often receive state payments as remuneration for their foster services; (b) placements in the homes of kinship caregivers with whom the child has a pre-existing relationship and who may also receive remuneration (but do not have to receive remuneration), as long as those placements involve contact with and coordination by CPS (i.e., excluding informal kinship care arrangements that do not involve, and are perhaps not known to, the state), and (c) placements in group homes or other institutional residential care settings, which may be state-owned or may be privately managed but contracted by CPS. Our analyses include all recorded placements in these categories.

The primary difference between national OOHC definitions concerns placements for older children, especially those aged 15-17. In Denmark, boarding school placements and several other non-institutional and non-foster family living arrangements are counted in a separate category but are often added into national OOHC estimates. Such children live outside the homes of their legal guardians or primary caregivers; however, their placements are overwhelmingly voluntary (and, in the case of boarding schools, are also frequent among the general population of Danish adolescents not in OOHC) and not de juris classified as OOHC. We excluded this category of placements from our analyses, since no corresponding categories exist in Australia and the US, and since children in comparable situations would not be counted as experiencing OOHC in those countries. This decision lowers observed rates of OOHC especially among Danish and Greenlander adolescents in Denmark aged 15–17 but closely aligns definitions of OOHC across countries.

Still, it was impossible to fully align measurements due to differences in administrative data collection. Danish authorities record single-year ages as of December 31; Statistics Greenland measures single-year age as of January 1; and AFCARS datasets measure single-year age as of October 1. Australian OOHC counts are computed for each national fiscal year (which begins on July 1); AFCARS counts are reported by US federal fiscal year (which begins on October 1); Danish OOHC counts are reported by calendar year; and Greenland OOHC are point-in-time counts as of January 1. We accepted these minor discrepancies as unavoidable compromises for cross-national comparisons. Some challenges to comparative analyses remained due to non-reporting by sub-national jurisdictions in Australia, the undercounting of tribally affiliated indigenous children in the US, and the differential reporting of children in pre-adoptive foster homes. We discuss these challenges below.

## Data and Methods

### Data

Our primary measure of interest is annual rates of OOHC among under-18 indigenous and non-indigenous populations. To compute these rates, we used counts of OOHC placements and corresponding population counts from national child welfare agencies and statistical agencies. The scope and structure of our data were in part shaped by data sovereignty considerations. After consultations with local indigenous researchers, we did not analyze data from New Zealand to be fully compliant with recommendations from the Māori Data Sovereignty Network, and we also did not analyze microdata from Australia (to which local indigenous communities have privileged access under the principles established at the 2018 Indigenous Data Sovereignty Summit), relying instead on aggregate annual OOHC counts. In Greenland, we consulted with indigenous researchers and worked extensively with local authorities to systematically collect data on OOHC for the first time; the data collection was funded by us, but the resulting datasets are owned and governed by Statistics Greenland.

#### Australia

Child welfare data are collected and maintained in the Child Protection National Minimum Dataset (CP-NMDS) by the Australian Institute of Health and Welfare (AIHW). AIHW uses child-level data submitted by states and territories to compute summary counts of OOHC for indigenous and non-indigenous populations during each fiscal year, which include (a) placements in the homes of non-kin foster families, (b) placements in the homes of kinship relatives, (c) placements in family group homes or other residential and institutional living facilities. National statistics exclude data from New South Wales, which does not transmit child-level data to AIHW.

Australian population counts are based on a combination of counts (for the total population) and mortality-adjusted projections (for indigenous populations) for June 30 of each calendar year by the Australian Bureau of Statistics. These population data, which are also used in official government publications, were provided by AIHW. We matched population counts to the coverage of OOHC counts by subtracting population counts from New South Wales from national counts.

#### United States

The primary US database for counting and tracking children in OOHC is the Adoption and Foster Care Analysis and Reporting System (AFCARS). Reporting into AFCARS is mandatory for all Child Protective Services (CPS) agencies that receive federal Title IV-E funding, which is a provision of the Social Security Act that provides federal matching funds to states to cover costs of foster care, guardianship assistance, and prevention services and functions as the primary mechanism through which federal funds are transferred to state and local child welfare agencies. Therefore, our analyses of OOHC in the US exclude indigenous children under the jurisdiction of non-federally funded tribal agencies. Unfortunately, very little is known about the total number of children placed in OOHC by tribal agencies without the involvement of a Title IV-E-funded agencies or about reporting differences between federally recognized tribes. This limitation, although not always acknowledged, is common to all studies that use AFCARS datasets to study race-specific patterns of OOHC.

Given this limitation, AFCARS then contains information on all children who spend more than 24 hours in continuous foster care in a given fiscal year, which includes (a) placements in the homes of non-kin foster families, (b) placements in the homes of kinship relatives, (c) placements in emergency shelters or residential care facilities, and (d) placements in pre-adoptive foster homes. Children in the latter category (temporary pre-adoptive foster placements) were historically also counted in Australian datasets but were excluded from national OOHC counts after 2018. We did not exclude such children because they are not consistently identifiable in AFCARS files, and because similar placements are likely to be counted as OOHC in Denmark and Greenland (making Australia the outlier).

We used AFCARS annual Foster Care Files to count the number of unique indigenous and non-indigenous children who experienced OOHC in a given fiscal year, i.e., children who newly entered OOHC as well as children who remained in OOHC after entering during a prior fiscal year. We used custom data tabulations from the US Census Bureau’s Population Estimates Program (PEP) to compute population denominators. These population estimates, which are provided annually to the National Data Archive on Child Abuse and Neglect (NDACAN) and model population counts for intercensal years by adjusting for births, deaths, and migration, have a distinct advance over commonly used age- and race-specific SEER population estimates that use bridged racial categories and assign a single race to all multiracial children. Since many AIAN children identify as multiracial, using bridged racial identifiers would have yielded artificially low AIAN population counts (i.e., our denominators) and thereby introduced upward bias into our estimates of indigenous OOHC rates. PEP racial identifiers allowed us to count all AIAN children regardless of whether they also identified as multi-racial and/or Hispanic. This aligned definitions of indigeneity across numerators and denominators.

#### Denmark

We constructed data on OOHC placements in Denmark from longitudinal administrative records on fostered children and youth (*Børn og Unge Anbragte Forløbsregister*, BUAF). BUAF contains records of all placements reported by Danish municipalities, including (a) placements in non-kin foster families, (b) placements with relatives, (c) placements in group homes and other residential living facilities. BUAF additionally includes data on boarding school and dormitory placements; however, we exclude these to increase the comparability of Danish OOHC estimates with those from other countries. BUAF also contains child-specific identifiers, which allowed us to identify indigenous children by linking their BUAF records to BEF (which contains information on parents and parental birthplace).

We also used BEF to compute Danish population counts. BEF includes all individuals with a registered Danish address as of December 31 and additionally contains demographic information, including single-year age and birthplace records.

#### Greenland

Greenlandic OOHC data were constructed specifically for this research project by Statistics Greenland based on addresses of children and parents from the Greenlandic population register, combined with municipality payment data on placement-related financial transfers. This made it possible to first identify children who did not reside with a parent and then determine if they lived (a) with close relatives (with remuneration), (b) in foster care, or (c) in an institutional residential facility. This left a residual category consisting of children who did not experience any of those living arrangements but also did not share an address with a parent (e.g., children living without renumeration with members of their extended families, children who had relocated from isolated settlements to larger communities for educational purposes, or children whose placements had been misclassified in administrative records). We addressed this by treating annual OOHC counts that included this residual category as a plausible upper bound, and by treating annual OOHC counts that excluded this residual category as a plausible lower bound. This approach reflected the assumption that some—but likely not all—placements in this residual category were substantively indistinguishable from OOHC. The resulting OOHC counts measure the number of unique children experiencing OOHC on January 1 of each calendar year.

We used age-specific population counts that were drawn from the Greenland Population Register and obtained through Statistics Greenland’s Data Bank.

### Methods

We performed three calculations. First, we combined raw annual counts of OOHC for indigenous and non-indigenous children with the corresponding population counts to estimate the unadjusted annual rates of OOHC in each country, computed separately for indigenous and non-indigenous children in four age buckets: 0-4 years, 5-9 years, 10-14 years, and 15-17 years. We used these age groups because single-year age data was unavailable in Denmark and Greenland due to privacy restrictions.

In some cases, data on indigeneity were incomplete due to missing data in child-level datasets (US, with 1.6% missing) or due to the suppression of low-n cells in aggregate datasets (Denmark, and Australia). In US datasets, we imputed missing values using Multiple Imputation by Chained Equations (MICE) with data on age, sex, state of residence, Hispanic ethnicity, other available racial identifiers (for multiracial children), and Temporary Assistance to Needy Families (TANF) eligibility. In Australian datasets, we used data on the single-year age distribution of OOHC placements to impute counts of indigenous children in a small number of cells that contained <4 observations and had been suppressed in accordance with disclosure avoidance rules. In Denmark, disclosure avoidance rules require the suppression of cell counts <5, meaning that a suppressed cell could contain anywhere from 1 to 4 observations. We were able to access restricted-use data on the distribution of suppressed counts and used these data to derive point estimates of annual counts and their corresponding confidence intervals.

Second, we modeled age-standardized OOHC rates with a parametric bootstrap approach. This model-based approach allowed us (a) to increase the comparability of estimates across countries by age-standardizing OOHC placement rates, (b) to propagate measurement and modeling uncertainties to our final estimates of OOHC rates, using 95% confidence intervals, and (c) to address concerns about the reliability of observed OOHC counts and of unadjusted annual rates that were computed from those counts. Annual counts can be quite low when indigenous children account for a small percentage of the total child population or when the total child population is itself small. As a result, data entry or classification errors can quickly bias annual rates and may also lead us to over-estimate inter-annual fluctuations in these rates.

To obtain model-based rates, we first sampled (using 1,000 iterations) from observed rates and their corresponding 95% CIs, assuming a normal distribution. We then fitted non-linear time trend models using Generalized Additive Models (GAMs) with smoothing splines, which determine optimal smoothness while capturing complex temporal patterns including rises, falls, and turning points. We incorporated model uncertainty (due to parameter uncertainty and residual variability) into our estimates by sampling from the fitted models’ prediction intervals. Finally, we age-standardized the modeled age-specific rates using the World Health Organization’s World Standard Population.

Third, we used observed and modeled rates to compute indigenous/non-indigenous disparities in OOHC. We computed those disparities by repeatedly sampling from probability distributions parameterized by indigenous and non-indigenous OOHC rate estimates and their associated 95% CIs, then divided the sampled OOHC rates of indigenous children by the sampled OOHC rates of non-indigenous children to obtain risk ratios for each country and year.

## Results

### Australia

**Figure 1.**
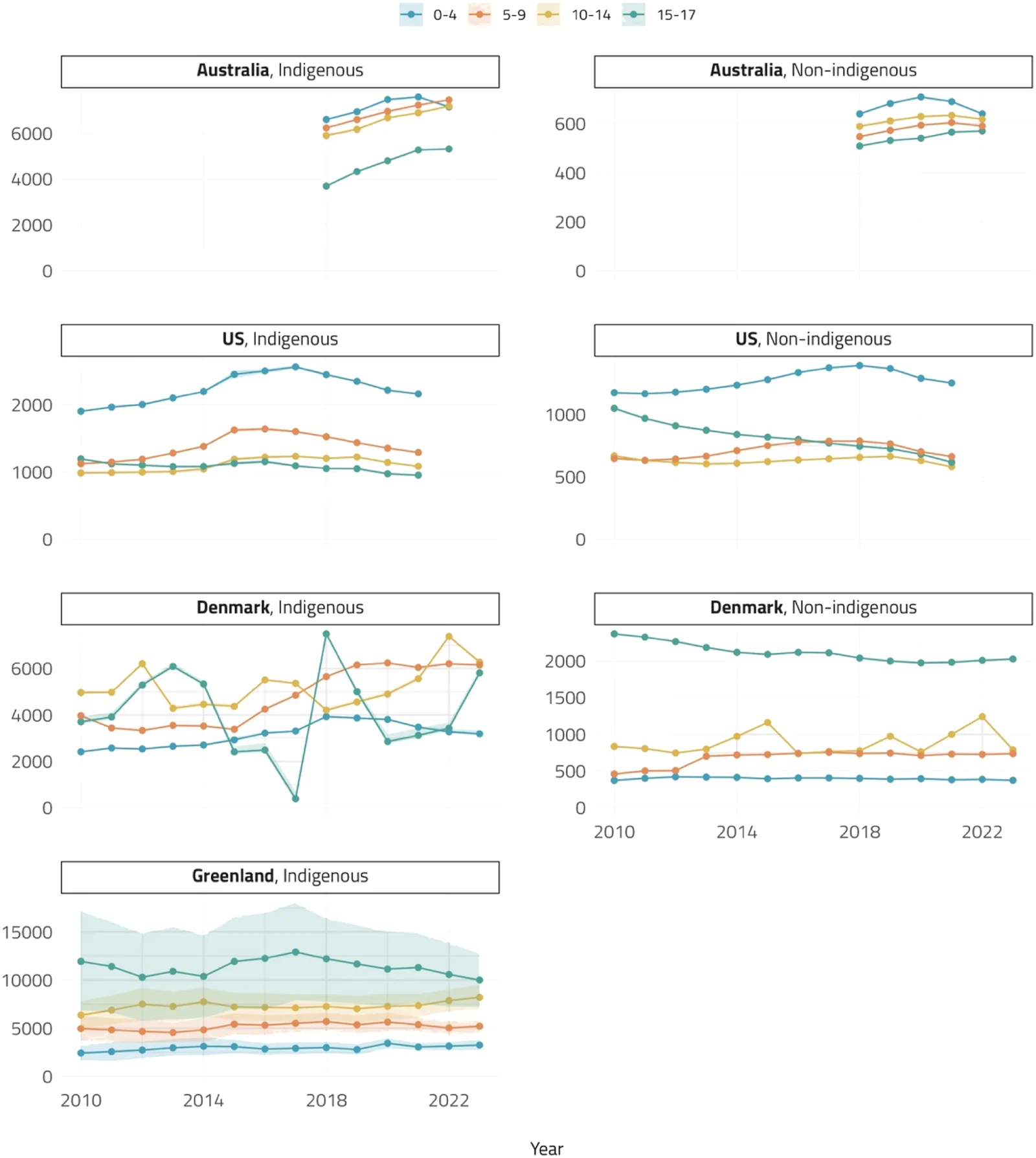
Observed annual rates of OOHC

In Australia, indigenous children experienced substantially higher rates of OOHC placement across all measured years compared to non-indigenous children. Observed annual rates among indigenous children ranged from 5,845 placements per 100,000 children in 2018 to 6948 placements per 100,000 in 2022, while non-indigenous children experienced OOHC rates of 579 per 100,000 in 2018 and 608 per 100,000 in 2022 (Figure 1).

**Figure 2.**
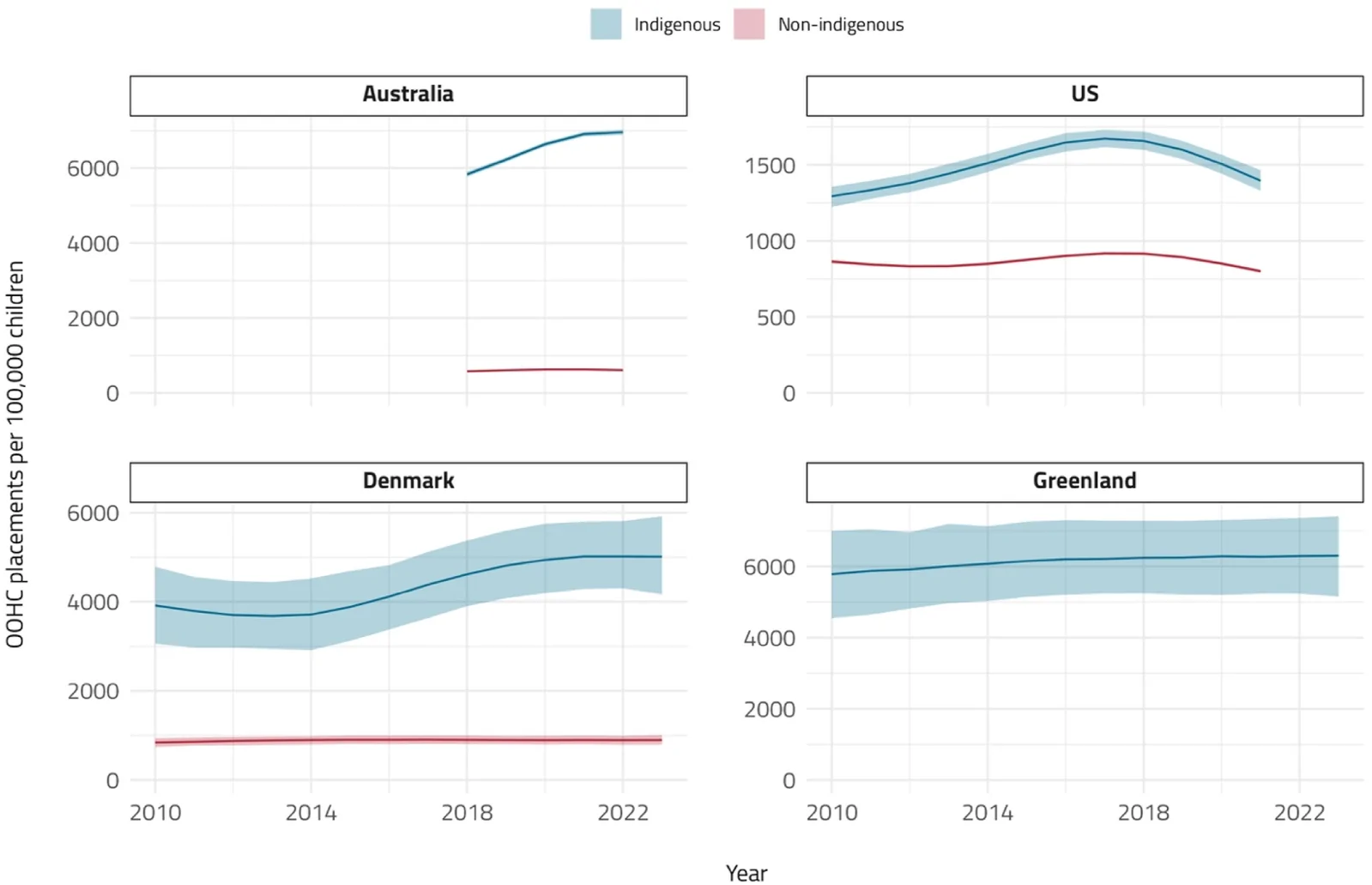
Modeled age-standardized annual rates of OOHC

Age-standardized modeled rates closely matched these observed rates, with indigenous OOHC rates ranging from 5829 per 100,000 (95% CI [5760, 5905]) in 2018 to 6958 per 100,000 (95% CI [6889, 7028]) in 2022 (Figure 2). OOHC rates were highest among children aged 0-4 and lowest among children aged 15–17 for indigenous and non-indigenous populations, but indigenous children had a slightly stronger age gradient.

These rates translate into high annual indigenous/non-indigenous placement risk ratios, ranging from 10.1 in 2018 to 11.4 in 2022. These were the highest ratios observed in any of the four countries studied (Table 1).

**Table 1.** Annual OOHC Placement Risk Ratios (Indigenous/Non-Indigenous) With Upper and Lower 95% CI Limits

Year	Australia		United States		Denmark	
	Observed	Modeled	Observed	Modeled	Observed	Modeled
2010			1.53 (1.52, 1.53)	1.5 (1.42, 1.58)	4.31 (4.25, 4.38)	4.65 (3.46, 5.95)
2011			1.6 (1.6, 1.6)	1.58 (1.5, 1.65)	4.23 (4.17, 4.28)	4.39 (3.42, 5.54)
2012			1.64 (1.64, 1.65)	1.66 (1.59, 1.73)	5.03 (5.01, 5.05)	4.24 (3.26, 5.3)
2013			1.71 (1.7, 1.71)	1.73 (1.66, 1.8)	4.4 (4.36, 4.45)	4.15 (3.24, 5.17)
2014			1.75 (1.75, 1.75)	1.78 (1.71, 1.85)	4.12 (4.07, 4.16)	4.13 (3.19, 5.14)
2015			1.92 (1.88, 1.96)	1.81 (1.75, 1.88)	3.36 (3.29, 3.43)	4.3 (3.42, 5.33)
2016			1.9 (1.87, 1.94)	1.82 (1.76, 1.89)	4.51 (4.47, 4.55)	4.54 (3.63, 5.55)
2017			1.88 (1.86, 1.9)	1.82 (1.76, 1.89)	4.18 (4.12, 4.23)	4.83 (3.88, 5.76)
2018	10.1 (10.08, 10.11)	10.06 (9.93, 10.19)	1.8 (1.79, 1.81)	1.81 (1.74, 1.87)	5.85 (5.82, 5.87)	5.15 (4.18, 6.27)
2019	10.24 (10.23, 10.25)	10.25 (10.13, 10.37)	1.77 (1.76, 1.78)	1.79 (1.72, 1.86)	5.23 (5.19, 5.27)	5.39 (4.46, 6.42)
2020	10.66 (10.66, 10.67)	10.56 (10.46, 10.67)	1.77 (1.76, 1.77)	1.77 (1.7, 1.85)	5.27 (5.21, 5.34)	5.53 (4.53, 6.67)
2021	11 (11, 11.01)	10.97 (10.85, 11.08)	1.81 (1.81, 1.82)	1.74 (1.65, 1.83)	5.04 (4.97, 5.1)	5.58 (4.54, 6.72)
2022	11.42 (11.42, 11.43)	11.41 (11.27, 11.54)			5.3 (5.23, 5.35)	5.64 (4.6, 6.72)
2023					6.16 (6.08, 6.24)	5.58 (4.52, 6.81)

### United States

American Indian/Alaska Native children experienced higher annual rates of OOHC than non-indigenous children. Observed annual rates among AIAN children (including multiracial and Hispanic AIAN children) increased from 1322 placements per 100,000 in 2010 to 1699 placements per 100,000 in 2016 before declining to 1419 placements per 100,000 in 2021. Non-indigenous placement rates for the same years were 867 per 100,000 (2010), 893 per 100,000 (2016), and 782 per 100,000 (2021). As in Australia, placement rates were highest for very young children aged 0-4.^1^

Age-standardized modeled estimates showed AIAN OOHC rates ranging from 1294 placements per 100,000 in 2010 (95% CI [1225, 1364]) to 1673 placements per 100,000 in 2017 (95% CI [1615, 1735]). Indigenous-to-non-indigenous placement ratios varied between 1.5 in 2010 and 1.9 in 2015–17. Disparities in OOHC have marginally declined in recent years, with our data showing risk ratios below 1.8 since 2019.

### Denmark

Children of Greenlandic ancestry in Denmark experienced markedly higher OOHC rates compared to the remaining Danish child population, as well as an increase in annual OOHC placement rates between 2010 and 2023. In 2010, Greenlander children living in Denmark had a placement rate of 3807 per 100,000, compared to 883 per 100,000 among non-indigenous children (i.e., children without Greenlander ancestry). In 2023, Greenlander children had a placement rate of 5482 per 100,000, compared to 891 per 100,000 among non-indigenous children. Age-standardized modeled rates for Greenlander children were 3922 per 100,000 in 2010 (95% CI [3050, 4761]) and 4994 per 100,000 in 2023 (95% CI [4172, 5809]). In contrast to Australia and the US, in Denmark children aged 15–17 had some of the highest OOHC placement rates, while children aged 0-4 had consistently the lowest OOHC placement rates. We return to this finding in the Conclusion.

The observed Danish rates imply OOHC placement risk ratios that sit between those observed in Australia and the US. In Denmark, indigenous children were around 4.3 times as likely as non-indigenous children to be in OOHC in 2010 and around 6.2 times as likely in 2023.

### Greenland

Given that the overwhelming majority of children in Greenland—and virtually all children in OOHC in Greenland—are indigenous, our analysis focused on overall OOHC rates rather than indigenous-to-non-indigenous comparisons. We found that annual OOHC placement rates in Greenland ranged from 5901 per 100,000 in 2010 to 6345 per 100,000 in 2017. Since then, annual rates have slightly declined to 6,175 placements per 100,000 in 2023. As in Denmark, annual rates are highest for older children (ages 15–17) and lowest for children aged 0-4.

Due to classificatory uncertainty—i.e., about the inclusion or exclusion of the residual category of “other” placement types in annual OOHC counts—and the relatively small size of the Greenlander child population, estimates of observed annual rates have wider confidence intervals than in other countries and are subject to interannual fluctuations. Age-standardized modeled rates provide more stable estimates, ranging from 5740 per 100,000 in 2010 (95% CI [4507, 6942]) to 6210 per 100,000 in 2017 (95% CI [5226, 7210]) and 6302 per 100,000 in 2023 (95% CI [5167, 7483]).

## Limitations

While our calculations incorporated uncertainty due to missing or suppressed indicators of indigeneity and due to ambiguous administrative classifications of OOHC in Greenland (i.e., uncertainty reflected in the numerators), our confidence intervals do not reflect measurement uncertainty in the population denominators, which were provided by national statistical agencies as annual point estimates without standard errors. This limitation was unavoidable.

Additionally, we emphasize a point we made in the footnote attached to the Results section: Different definitions of indigeneity (e.g., excluding multiracial children and/or Hispanic children or including Native Hawaiian/Pacific Islander children in the US, or requiring both parents to have Greenlander ancestry in Denmark) affect observed OOHC rates and ratios. Those choices are particularly consequential in the US, where definitions of indigeneity vary across government agencies and research teams and where salient choices—about the inclusion of multiracial persons and/or Hispanics—have to be made. Our main analyses used an expansive definition of indigeneity (which recognizes self-identified multiracial individuals as part of the indigenous community) while the attached footnote presented results obtained under a more restrictive definition (which was the dominant definition based on self-reported race until the 2000 U.S. Census), but we take no position on any particular definition being “correct.” Instead, we note that the resulting differences in our OOHC estimates provide suggestive evidence that child welfare contact is unevenly distributed among children who could plausibly identify as indigenous, and is concentrated among single-race and non-Hispanic AIAN children for whom indigeneity has historically been the only or primary racial identity (Snipp, 1986). We strongly encourage future research in this domain, which fits with a recent emphasis of inequality researchers on subgroups within and across official categories (Monk, 2022).

Finally, we reiterate an unavoidable limitation due to the way AFCARS data are collected in the US: Tribal welfare authorities who do not receive federal funding do not report into the AFCARS system, and the number of indigenous children who are thereby uncounted in administrative records despite experiencing OOHC within their respective tribal communities is unknown. To our knowledge, this number has never been reliably estimated.

## Conclusion

The historical removal of indigenous children and their continued over-representation in foster care is well-established (Dvalishvili et al., 2024; Jacobs, 2014; Krakouer et al., 2018; Thorleifsen et al., 2020; Yi et al., 2020). However, specific knowledge of contemporary OOHC patterns remains limited, especially in Greenland and Denmark. Our analyses update estimates of indigenous OOHC rates in some countries (US and Australia) and provide the first-ever estimates of such rates in others (Denmark and Greenland). They highlight persistently high annual rates of OOHC among indigenous children—broadly speaking, around 6% of indigenous children in Australia and Greenland, around 4.5% of indigenous children in Denmark, and around 1.5% of indigenous children in the US are in OOHC in any given year. Focusing on annual risk ratios, we showed that indigenous children were significantly more likely than non-indigenous children to experience OOHC in any given year, with ratios highest in Australia. Data from AIHW suggest that the particularly high annual incidence of OOHC among indigenous children (and the correspondingly large risk ratios) may in part be a consequence of long stints in foster care: As of 2021-2022, 45% of indigenous children in OOHC had been continuously placed for five years or more (Australian Institute of Health and Welfare, 2025b).

Our findings also showed that OOHC placement rates vary across age groups—but that age patterns are not consistent across countries. In Australia and the US, very young indigenous children are generally at a greater risk of experiencing OOHC in any given year, whereas older children are at a greater risk in Denmark and Greenland. This is likely a consequence of different policy environments. For example, elevated placement rates in the 15-17 age group in Denmark and Greenland are consistent with local authorities’ use of OOHC as a preventive measure against adolescent risk behaviors, such as juvenile criminal activity.

These findings suggest two avenues for research. First, they highlight the importance of ethically responsible research into the causes of indigenous over-representation in child welfare systems, especially in countries where the potentially long shadow of settler colonialism has received less scholarly and political attention. Such over-representation could be due to a greater prevalence of underlying risk factors (e.g. risk factors related to poverty) in minority communities or due to the biased decision-making of child welfare officials, both of which have been extensively studied for Black populations in the US (Baron et al., 2024; Drake et al., 2009, 2011, 2023; Fong, 2020). Second, our findings suggest the need for studies that can follow indigenous populations from youth into adulthood to estimate the impact of OOHC on subsequent life course trajectories. The short- and long-term outcomes of OOHC are actively debated (Clemens et al., 2018; Courtney & Hook, 2017; Doyle, 2013; Dvalishvili et al., 2024; Eiermann et al., 2025; Gross & Baron, 2022; Zlotnick et al., 2012), with some studies finding that the effects of childhood adversity can persist for a long time (Jaffee, 2017; Li et al., 2019; Stansfeld et al., 2017). However, most relevant studies do not specifically analyze indigenous populations due to research design decisions or limited sample sizes. Especially when the population of interest is relatively small or likely undercounted in administrative data, advancing population-specific knowledge may require targeted data collection and research designs that specifically center the experiences of indigenous youths and adults. Yet a better understanding of how OOHC affects the magnitude and persistence of adversity-induced vulnerabilities among indigenous populations would close an important knowledge gap and additionally has the potential to inform OOHC policy designs.

## Data Availability

AIHW does not allow for archiving of Australian data, but similar data as used in this article can be obtained by request to AIHW. Aggregate-level data for Denmark, Greenland, and the US is available on the Open Science Framework: https://osf.io/p56xa.

## Note

1. In supplemental analyses, we replaced our primary expansive definition of indigeneity (all AIAN children, including multiracial and Hispanic children) with a more restrictive definition (Single-race, non-Hispanic AIAN children only). We also replaced our primary population denominators with alternative denominators—also using data from the Census Bureau’s Population Estimates Program—that match the more restrictive definition. These changes increase both the observed annual rates of OOHC and the indigenous/non-indigenous ratios of OOHC. Using the alternative definition, we found that indigenous OOHC rates evolved from 1995 placements per 100,000 in 2010 to 2447 placements per 100,000 in 2021. The indigenous placement rate was highest in 2018 at 2629 per 100,000. In other words, we observe a similar overall upward trend and a similar peak in OOHC placements between 2016 and 2019, but we also note that annual rates are 40-53% higher under this alternative definition. Observed annual indigenous/non-indigenous ratios based on this definition vary from 2.3 (in 2010) to 3.0 (in 2021). We take no position on any definition being “correct” but note that the more restrictive definition—which yields higher estimates of annual rates and racial disparities—tends to pick up individuals for whom indigeneity is the main or primary marker of identity (Liebler, 1996; Snipp, 1986), while the more expansive definition—which has been used in recent research and yields lower estimates of annual rates and racial disparities—also picks up individuals who have complex multi-racial and/or multi-ethnic identities.

## References

[bibr1-10775595251411524] Australian Institute of Health and Welfare . (2025a). Child protection Australia 2022–23. Australian Institute of Health and Welfare. https://www.aihw.gov.au/reports/child-protection/child-protection-australia-2022-23/

[bibr2-10775595251411524] Australian Institute of Health and Welfare . (2025b). Closing the gap targets: Key findings and implications. Australian Institute of Health and Welfare. 10.25816/GQTF-2K57

[bibr3-10775595251411524] Baron E. J. Doyle J. J. Emanuel N. Hull P. Ryan J. (2024). Discrimination in multiphase systems: Evidence from child protection. Quarterly Journal of Economics, 139(3), 1611–1664. 10.1093/qje/qjae007 PMC11772066 39872361

[bibr4-10775595251411524] Brown H. E. (2020). Who is an Indian child? Institutional context, tribal sovereignty, and race-making in fragmented States. American Sociological Review, 85(5), 776–805. 10.1177/0003122420944165

[bibr5-10775595251411524] Clemens E. V. Klopfenstein K. Lalonde T. L. Tis M. (2018). The effects of placement and school stability on academic growth trajectories of students in foster care. Children and Youth Services Review, 87, 86–94. 10.1016/j.childyouth.2018.02.015

[bibr6-10775595251411524] Courtney M. E. Hook J. L. (2017). The potential educational benefits of extending foster care to young adults: Findings from a natural experiment. Children and Youth Services Review, 72, 124–132. 10.1016/j.childyouth.2016.09.030

[bibr7-10775595251411524] Doyle J. J. (2013). Causal effects of foster care: An instrumental-variables approach. Children and Youth Services Review, 35(7), 1143–1151. 10.1016/j.childyouth.2011.03.014

[bibr8-10775595251411524] Drake B. Jolley J. M. Lanier P. Fluke J. Barth R. P. Jonson-Reid M. (2011). Racial bias in child protection? A comparison of competing explanations using national data. Pediatrics, 127(3), 471–478. 10.1542/peds.2010-1710 PMC9923773 21300678

[bibr9-10775595251411524] Drake B. Jones D. Kim H. Gyourko J. Garcia A. Barth R. P. Font S. A. Putnam-Hornstein E. Duerr Berrick J. Greeson J. K. P. Cook V. Kohl P. L. Jonson-Reid M. (2023). Racial/Ethnic differences in child protective services reporting, substantiation and placement, with comparison to Non-CPS risks and outcomes: 2005–2019. Child Maltreatment, 28(4), 683–699. 10.1177/10775595231167320 36990447

[bibr10-10775595251411524] Drake B. Lee S. M. Jonson-Reid M. (2009). Race and child maltreatment reporting: Are blacks overrepresented? Children and Youth Services Review, 31(3), 309–316. 10.1016/j.childyouth.2008.08.004 PMC5400107 28435177

[bibr11-10775595251411524] Dvalishvili D. Jonson-Reid M. Drake B. (2024). Childhood poverty and foster care placement: Implications for practice and policy. Child Abuse & Neglect, 154, Article 106926. 10.1016/j.chiabu.2024.106926 PMC11316657 38964010

[bibr12-10775595251411524] Edwards F. Rocha Beardall T. Curtis H. (2023). American Indian and Alaska Native overexposure to foster care and family surveillance in the US: A quantitative overview of contemporary system contact. Children and Youth Services Review, 149, Article 106915. 10.1016/j.childyouth.2023.106915

[bibr13-10775595251411524] Edwards F. Wakefield S. Healy K. Wildeman C. (2021). Contact with Child Protective Services is pervasive but unequally distributed by race and ethnicity in large US counties. Proceedings of the National Academy of Sciences of the United States of America, 118(30), Article e2106272118. 10.1073/pnas.2106272118 PMC8325358 34282022

[bibr14-10775595251411524] Eiermann M. Baker G. Wildeman C. J. (2025). Discordant patterns and adulthood consequences of childhood maltreatment and foster care placement. Social Forces, soaf077. 10.1093/sf/soaf077 PMC13179106 42146771

[bibr15-10775595251411524] Fong K. (2020). Getting eyes in the home: Child protective services investigations and State surveillance of family life. American Sociological Review, 85(4), 610–638. 10.1177/0003122420938460

[bibr16-10775595251411524] Gross M. Baron E. J. (2022). Temporary stays and persistent gains: The causal effects of Foster care. American Economic Journal: Applied Economics, 14(2), 170–199. 10.1257/app.20200204

[bibr17-10775595251411524] Jacobs M. D. (2014). A generation removed: The fostering and adoption of Indigenous children in the postwar world. U of Nebraska Press.

[bibr18-10775595251411524] Jaffee S. R. (2017). Child maltreatment and risk for psychopathology in childhood and adulthood. Annual Review of Clinical Psychology, 13(1), 525–551. 10.1146/annurev-clinpsy-032816-045005 28375720

[bibr19-10775595251411524] Jensen B. (2021). Barndomshjem eller børnehjem? Et kvalitativt studie af 38 børns oplevelser af at blive og være anbragt på døgninstitution i Grønland [Ph.D. thesis]. Ilisimatusarfik.

[bibr20-10775595251411524] Krakouer J. Wise S. Connolly M. (2018). “We live and breathe through culture”: Conceptualising cultural connection for Indigenous Australian children in out-of-home care. Australian Social Work, 71(3), 265–276. 10.1080/0312407X.2018.1454485

[bibr21-10775595251411524] Li L. Pereira S. M. P. Power C. (2019). Childhood maltreatment and biomarkers for cardiometabolic disease in mid-adulthood in a prospective British birth cohort: Associations and potential explanations. BMJ Open, 9(3), Article e024079. 10.1136/bmjopen-2018-024079 PMC6475361 30904846

[bibr22-10775595251411524] Liebler C. A. (1996). American Indian ethnic identity: An analysis of tribal specification in the 1990 census (No. CDE working paper no. 96-20). Center for Demography and Ecology, University of Wisconsin-Madison. 12233488

[bibr23-10775595251411524] Monk E. P. (2022). Inequality without groups: Contemporary theories of categories, intersectional typicality, and the disaggregation of difference. Sociological Theory, 40(1), 3–27. 10.1177/07352751221076863

[bibr24-10775595251411524] Nobles M. (2000). Shades of citizenship: Race and the census in modern politics. Stanford University Press.

[bibr25-10775595251411524] Ring I. Brown N. (2003). The health status of indigenous peoples and others. BMJ, 327(7412), 404–405. 10.1136/bmj.327.7412.404 PMC188480 12933706

[bibr26-10775595251411524] Sinclair R. (2007). Identity lost and found: Lessons from the sixties scoop. First Peoples Child and Family Review, 3(1), 65–82. 10.7202/1069527ar

[bibr27-10775595251411524] Snipp C. M. (1986). Who are American Indians? Some observations about the perils and pitfalls of data for race and ethnicity. Population Research and Policy Review, 5(3), 237–252. 10.1007/BF00136786

[bibr28-10775595251411524] Snipp C. M. (2003). Racial measurement in the American census: Past practices and implications for the future. Annual Review of Sociology, 29(1), 563–588. 10.1146/annurev.soc.29.010202.100006

[bibr29-10775595251411524] Stanley E. de Froideville S. M. (2020). From vulnerability to risk: Consolidating state interventions towards Māori children and young people in New Zealand. Critical Social Policy, 40(4), 526–545. 10.1177/0261018319895203

[bibr30-10775595251411524] Stansfeld S. A. Clark C. Smuk M. Power C. Davidson T. Rodgers B. (2017). Childhood adversity and midlife suicidal ideation. Psychological Medicine, 47(2), 327–340. 10.1017/S0033291716002336 PMC5216460 27762177

[bibr31-10775595251411524] Thorleifsen D. Jensen E. L. Nexø S. A. (with danmark Social-og Ældreministeriet) (2020). Historisk udredning om de 22 grønlandske børn der blev sendt til Danmark i 1951: Afgivet den 15. November 2020. Social- og Ældreministeriet.

[bibr32-10775595251411524] Tilbury C. (2009). The over-representation of indigenous children in the Australian child welfare system. International Journal of Social Welfare, 18(1), 57–64. 10.1111/j.1468-2397.2008.00577.x

[bibr33-10775595251411524] Trocmé N. Knoke D. Blackstock C. (2004). Pathways to the overrepresentation of Aboriginal children in Canada’s child welfare System. Social Service Review, 78(4), 577–600. 10.1086/424545

[bibr34-10775595251411524] Tróndheim G. (2010). Grønlandsk adoption—Et vigtigt element i grønlandsk slægtskab. Social Kritik, 22(2010), 123.

[bibr35-10775595251411524] Valentine B. Gray M. (2006). Keeping them home: Aboriginal out-of-home care in Australia. Families in Society: The Journal of Contemporary Social Services, 87(4), 537–545. 10.1606/1044-3894.3569

[bibr36-10775595251411524] VIVE . (2022). Børn med grønlandsk baggrund, der er anbragt i Danmark—Fra danske kommuner. VIVE.

[bibr37-10775595251411524] Wildeman C. Roehrkasse A. F. Gibbons A. Sernaker S. Becker L. Fallesen P. (2025). Two decades of child welfare System contact in the global north: A research note on trends in 44 countries. Demography, 62(1), 1–15. 10.1215/00703370-11793609 PMC12165327 39898618

[bibr38-10775595251411524] Yi Y. Edwards F. R. Wildeman C. (2020). Cumulative prevalence of confirmed maltreatment and Foster care placement for US children by Race/Ethnicity, 2011–2016. American Journal of Public Health, 110(5), 704–709. 10.2105/AJPH.2019.305554 PMC7144424 32191517

[bibr39-10775595251411524] Zlotnick C. Tam T. W. Soman L. A. (2012). Life course outcomes on mental and physical health: The impact of Foster care on adulthood. American Journal of Public Health, 102(3), 534–540. 10.2105/AJPH.2011.300285 PMC3487656 22390519

